# Graph Neural Network Model for Prediction of Non-Small Cell Lung Cancer Lymph Node Metastasis Using Protein–Protein Interaction Network and ^18^F-FDG PET/CT Radiomics

**DOI:** 10.3390/ijms25020698

**Published:** 2024-01-05

**Authors:** Hyemin Ju, Kangsan Kim, Byung Il Kim, Sang-Keun Woo

**Affiliations:** 1 Radiological and Medico-Oncological Sciences, University of Science and Technology, Daejeon 34113, Republic of Korea; hmju@kirams.re.kr; 2 Division of RI-Convergence Research, Korea Institute of Radiological and Medical Sciences, Seoul 07812, Republic of Korea; krmount@kirams.re.kr; 3 Department of Nuclear Medicine, Korea Institute of Radiological and Medical Sciences, Seoul 07812, Republic of Korea; kimbi@kirams.re.kr

**Keywords:** radiogenomics, ^18^F-FDG PET, CT, NSCLC, protein–protein interaction, GNN

## Abstract

The image texture features obtained from ^18^F-fluorodeoxyglucose positron emission tomography/computed tomography (^18^F-FDG PET/CT) images of non-small cell lung cancer (NSCLC) have revealed tumor heterogeneity. A combination of genomic data and radiomics may improve the prediction of tumor prognosis. This study aimed to predict NSCLC metastasis using a graph neural network (GNN) obtained by combining a protein–protein interaction (PPI) network based on gene expression data and image texture features. ^18^F-FDG PET/CT images and RNA sequencing data of 93 patients with NSCLC were acquired from The Cancer Imaging Archive. Image texture features were extracted from ^18^F-FDG PET/CT images and area under the curve receiver operating characteristic curve (AUC) of each image feature was calculated. Weighted gene co-expression network analysis (WGCNA) was used to construct gene modules, followed by functional enrichment analysis and identification of differentially expressed genes. The PPI of each gene module and genes belonging to metastasis-related processes were converted via a graph attention network. Images and genomic features were concatenated. The GNN model using PPI modules from WGCNA and metastasis-related functions combined with image texture features was evaluated quantitatively. Fifty-five image texture features were extracted from ^18^F-FDG PET/CT, and radiomic features were selected based on AUC (n = 10). Eighty-six gene modules were clustered by WGCNA. Genes (n = 19) enriched in the metastasis-related pathways were filtered using DEG analysis. The accuracy of the PPI network, derived from WGCNA modules and metastasis-related genes, improved from 0.4795 to 0.5830 (*p* < 2.75 × 10^−12^). Integrating PPI of four metastasis-related genes with ^18^F-FDG PET/CT image features in a GNN model elevated its accuracy over a without image feature model to 0.8545 (95% CI = 0.8401–0.8689, *p*-value < 0.02). This model demonstrated significant enhancement compared to the model using PPI and ^18^F-FDG PET/CT derived from WGCNA (*p*-value < 0.02), underscoring the critical role of metastasis-related genes in prediction model. The enhanced predictive capability of the lymph node metastasis prediction GNN model for NSCLC, achieved through the integration of comprehensive image features with genomic data, demonstrates promise for clinical implementation.

## 1. Introduction

Lung cancer accounts for the majority of cancer cases worldwide, and non-small cell lung cancer (NSCLC) is the predominant type of lung cancer [1]. Metastasis is a critical factor in disease progression and can significantly affect the treatment options and prognosis. Approximately 40% of patients with lung cancer develop metastatic disease [2,3]. According to the statistics adapted from the American Cancer Society for regional NSCLC, which is characterized by the spread of cancer cells from the lungs to the surrounding lymph nodes, the 5-year relative survival rate is approximately 37%.

Radiomics is a technique that involves the analysis of medical images to extract various tumor characteristics [4]. The radiomics extraction process can help to quantify tumor heterogeneity [5]. NSCLC exhibits tumor heterogeneity [6], which arises from various factors within the tumor, such as different cell types, shapes, and metabolic activities. Radiomics allows the quantification and visualization of this heterogeneity and reveals differences among various parts of the tumor [7]. Information obtained through radiomic analysis can be valuable for predicting tumor prognosis, evaluating treatment responses, making treatment plans, and determining personalized treatment strategies [8]. Thus, radiomics is an important tool for detecting and understanding tumor heterogeneity.

A protein–protein interaction (PPI) network is a graphical representation of the physical and functional interactions between proteins within a biological system [9]. It helps in understanding how proteins collaborate and communicate within a cell or organism [10]. PPI networks are crucial for understanding complex cellular processes [11], signaling pathways [12], and molecular basis of various diseases [13]. By annotating PPI interactions, the processes underlying tumor activities can be understood and potential therapeutic targets for diverse medical conditions can be identified [14]. Engin et al. built a network based on PPI and identified gene mutations associated with breast cancer metastasis to the brain and lungs [15]. Liu et al. built a cancer-specific gene network from PPI, mined the network structure properties, and predicted cancer-causing genes by combining biological information, such as the mutation frequency and differential gene expression, thus showing that the network-based features included mutation frequency and genetic differential expression [16]. In a study by Yu-Dong Cai et al., a large-scale network was constructed using PPI, and hub genes that could mediate breast cancer metastasis to the bone were identified through functional analysis; furthermore, permutation false discovery rate (FDR), betweenness ratio, and maximum-minimum interaction score were used to determine whether these genes were involved in metastasis [17]. Given the rich insights provided by PPI networks into the roles of genes in cancer progression and metastasis, we intended to harness this resource to develop a predictive network for NSCLC metastasis.

Graph neural network (GNN) is a type of deep learning model for processing and analyzing graph-structured data [18]. GNN utilizes the hierarchical structure of graphs, and comprises nodes and edges [19]. GNN involves multiple layers where node features are updated through the aggregation of information from the node and its neighbors using weights and activation functions [20]. This process of information propagation helps in identifying node interaction and yields information regarding the relationships and patterns within the graph’s structure [21]. GNN has powerful feature extraction [22] and relationship learning capabilities, which are ideal for handling the complexity of graph data [23]. This allows GNN to perform various tasks in different domains, leading to significant advances in molecular biology [24,25,26]. This study utilized a GNN model that integrated a PPI network describing connections between genes and cancer characteristics with radiomics data that capture the variability within tumor images to predict metastatic progression in NSCLC.

## 2. Results

### 2.1. Gene and Image Texture Feature Selection

**Figure 1:**
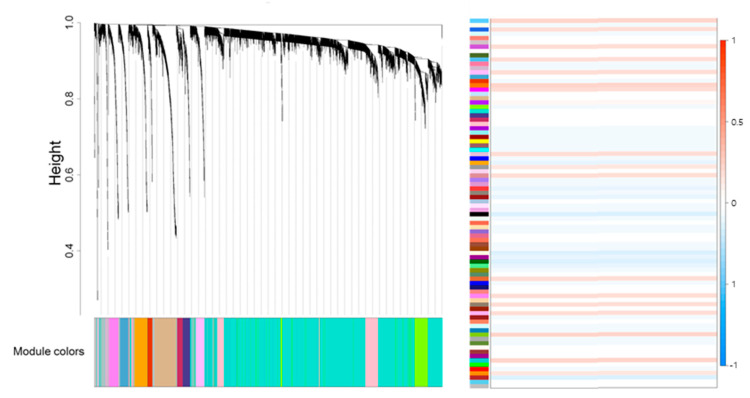
Weighted gene co-expression network analysis. A dendrogram illustrating 86 modules, where genes are clustered based on dissimilarity, highlighted with a color band showing results from an automatic single-block analysis (**left**); Associations between gene modules and lymph node metastasis traits were assessed by examining the correlations between the eigengenes of these modules and the traits. Each block in the heatmap represents a different module and modules were enriched with clustered genes. The intensity of the color in each block reflects the strength of the correlation between the module and the lymph node metastasis trait in non-small cell lung cancer (**right**). The intensity of the color in each block reflects the strength of the correlation between the module and the lymph node metastasis trait in non-small cell lung cancer Sky Blue 1 and Yellow Green modules were selected for functional analysis due to their significant correlations, evidenced by *p*-values of 0.03 and 0.047, respectively.

The co-expressed genes were grouped into 86 modules. Except for the modules for which no PPI network was formed, the genes in the 73 modules were used to construct the PPI (Figure 1). The process used in this study is schematically shown in Figure S1. In WGCNA analysis, significant correlations between lymph node metastasis and the Sky Blue 1 and Yellow Green modules were observed. The Sky Blue 1 module, with a *p*-value of 0.03, and the Yellow Green module, with a *p*-value of 0.047, both demonstrated notable associations. Functional analysis was subsequently conducted on these modules, encompassing 74 genes from the Sky Blue 1 module and 127 genes from the Yellow Green module. Thirty-four genes in each module were enriched in various metastatic pathways, including “HIF-1 signaling”, “Central carbon metabolism in cancer”, “Focal adhesion pathway”, and “Ubiquitin” (*p* < 0.05). Nineteen enriched DEGs were selected. After univariate analyses, 12 genes were obtained (*p* < 0.05). Five genes (EGF, HKDC1, PIK3R1, MYLK4, and COL6A5) were selected based on their GS scores.

**Figure 2:**
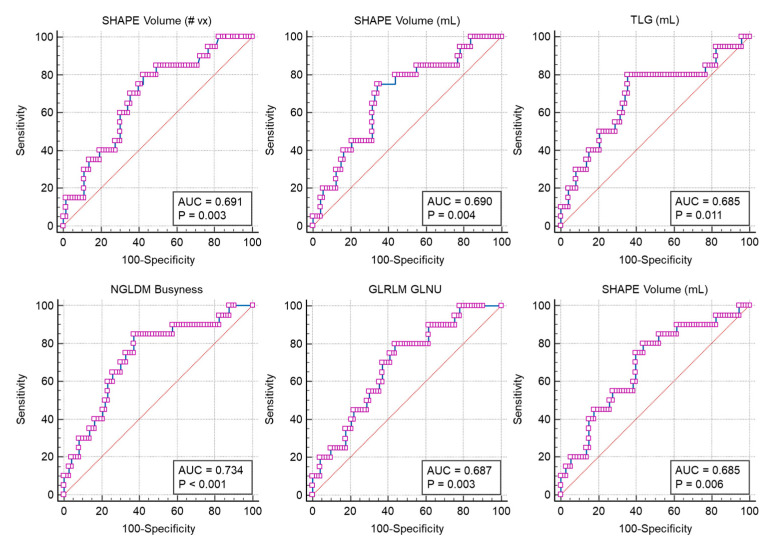
The receiver operating characteristic curve of six image features, comprising three attributes each from ^18^F-FDG PET and CT images, which have demonstrated the highest area under the curve receiver operating characteristic curve values. *p*-values were calculated based on the Hanley and McNeil standard error formula.

Image texture features with AUC of 0.68 or higher were selected from ^18^F-FDG PET (TLG, SHAPE Volume [mL], SHAPE Volume [# vx], SHAPE Compacity only, GLRLM GLNU, GLZLM LZHGE), and CT images (SHAPE Volume [mL], GLRLM GLNU, NGLDM Busyness, GLZLM_GLNU) (Figure 2).

### 2.2. GNN Model

In this study, the model was assessed based on genes associated with four key metastasis-related functions. The GNN model was constructed using the 73 gene modules obtained using WGCNA. Only DEGs identified from this gene set were used for further evaluation. Subsequent analysis was refined to include only 12 DEGs identified in the univariate analysis. Additionally, based on WGCNA outcomes, genes exhibiting elevated GS scores were employed for model assessment.

The PPI network, formulated from metastasis-related genes, achieved an accuracy of 0.5830, significantly exceeding the 0.4795 accuracy of the GNN model that incorporated a PPI network derived from WGCNA modules (*p*-value < 2.75 × 10^−12^). The integration of PPI from four metastasis-related genes with ^18^F-FDG PET/CT imaging features resulted in a substantial accuracy boost to 0.8545 (95% confidence interval [CI] = 0.8401–0.8689) compared to the model without image (*p*-value < 0.02). These findings emphasize the crucial impact of metastasis-related genes on the predictive model, demonstrating a superior accuracy compared to models employing PPI and ^18^F-FDG PET/CT image features derived from WGCNA generated modules (*p*-value < 0.02). This improvement was notably more pronounced than in the model combining PPI and ^18^F-FDG PET/CT from WGCNA (*p*-value < 0.02), highlighting the pivotal impact of metastasis-related genes in the predictive model.

The combined ^18^F-FDG PET/CT image features consistently yielded higher AUC values across various methods than individual ^18^F-FDG PET or CT image features. This indicates that the use of both imaging modalities improves the predictive accuracy of the model. Without incorporating any image features, the accuracy and was recorded at 0.5830. However, when incorporating image features, the accuracy values improved: 0.8143 (95% CI = 0.8051–0.8234) with ^18^F-FDG PET scans, 0.7955 (95% CI = 0.7808–0.8101) with CT scans, and the highest at 0.8545 (95% CI = 0.8401–0.8689) when combining ^18^F-FDG PET with CT scans. The model’s performance was generally higher when using whole image features as compared to when using selected image features with AUC ≥ 0.68. The findings suggest that incorporating a broader range of image factors enhances the performance of the models. This implies that the use of a comprehensive set of image features enhances the predictive ability of the model. The model demonstrated optimal performance when the ^18^F-FDG PET and CT imaging data were integrated, yielding the highest level of accuracy observed in the study. The results reinforce the notion that a multifaceted approach, combining diverse data types, holds promise for improving model predictions in the context of deep learning applications. This underscores the significance of integrating genomic data with imaging features, particularly those derived from multiple imaging modalities, to optimize predictive accuracy.

Univariate analysis and GS score criteria seemed to reduce the model’s performance compared to the use of all genes involved in the four metastasis functions. This suggests that certain genes carry more weight or significance in predicting outcomes (Table 1). The enhanced predictive capacity of the GNN model for lymph node metastasis in NSCLC, through the amalgamation of extensive imaging features and genomic data shows significant potential for clinical application.

## 3. Discussion

In this study, we employed a GNN model to investigate the interplay between genomic and imaging features for predicting lymph node metastasis in NSCLC. To ensure the reliability and validity of our model, we extensively analyzed its performance across a spectrum of genomic criteria and imaging techniques. The model showed reliable performance across different genomic criteria and imaging modalities development of a novel GNN model capable of noninvasively predicting lymph node metastasis in NSCLC. This advancement is particularly noteworthy in the context of personalized medical care. Our model opens up new avenues for tailoring treatment strategies to individual patient needs, a step that is crucial in the fight against NSCLC. By enabling earlier and more accurate detection of lymph node metastasis, our model holds the promise of significantly improving patient outcomes and optimizing treatment approaches.

These results underscore the significance of integrating image features extracted from ^18^F-FDG PET and CT. The GNN model tended to have a higher accuracy and AUC for predicting lymph node metastasis using image features extracted from ^18^F-FDG PET and CT together than when they were used separately. When the model utilized both ^18^F-FDG PET and CT features concurrently, it achieved the highest accuracy (0.8545), indicating the synergistic value of combining these modalities for the GNN model. The GNN model performed poorly in terms of prediction in the absence of image data, highlighting the power of image data to predict the lymph node metastases of NSCLC.

Radiogenomics facilitates the structural delineation of lesions and offers insights into the functional aspects of tumor, encompassing their biological characteristics [27]. Radiogenetics is a measure of how well imaging factors extracted from inside a tumor reflect the characteristics of the tumor. Therefore, we demonstrated that image characteristics and gene expression levels extracted from NSCLC are factors predicting metastasis. Upon closer examination, gene-based evaluation, especially based on WGCNA with 73 modules, highlighted the intricate genomic interactions that the GNN model can exploit. Specifically, it yielded accuracy values ranging from 0.8143 (^18^F-FDG PET) to 0.7955 (CT) with genetic imaging features mapped to four metastasis-related features, further confirming the model’s ability to identify genomic patterns.

Furthermore, the use of DEGs and genes filtered through univariate analysis bolstered the predictive capacity of the model. This indicates that a targeted approach focusing on genes with heightened expression variances or significant univariate associations can enhance model performance. Our findings also underscore the potential of GS derived from WGCNA. The AUC value of the model with genes with high GS scores provides compelling evidence of their predictive potency. Using a statistical approach, we were able to evaluate models that showed high prediction rates. Kirienko et al. demonstrated that the performance of a machine learning model that predicts the recurrence of lung cancer was improved by using radiogenomics, which combines radiomics and genomic data [28]. Zhou et al. developed a radiogenomic map linking gene expression profiles with CT image features, which highlighted the associations between CT characteristics and metagenes representing canonical molecular pathways [29]. Li et al. proposed a lung adenocarcinoma multi-classification model based on a GNN model using radiomics data extracted from the region of interest (ROI) of CT images [30].

The AUC is an effective measure for assessing how well a variable, such as an image feature, can differentiate between distinct classes. This metric is particularly crucial in classification scenarios aimed at distinguishing between different outcomes accurately. AUC values range from 0.5, indicating no predictive power, to 1, which represents perfect prediction. A higher AUC value suggests a greater capability of the variable in accurately classifying the outcomes. The choice of 0.68 as the threshold, though somewhat subjective, is strategic. Set slightly below the more rigorous standard of 0.7, this threshold aims to balance the model’s precision with the inclusion of variables that could be advantageous. This decision helps in reducing the likelihood of overfitting and bias, which might arise from using variables with excessively high AUC values. Further analysis revealed that thresholds set significantly higher than 0.68 led to challenges in model construction. Specifically, higher thresholds resulted in too few variables, diminishing the model’s predictive power. Thus, the threshold of 0.68 was meticulously chosen to optimize the number of variables. This approach ensures the model has enough predictive strength maintaining a balance crucial for a robust and effective predictive model.

The integration of radiogenomic data, combining both imaging and genomic information, represents a significant advancement in the field. By correlating imaging features with genomic data, the study bridges the gap between macroscopic imaging findings and the underlying microscopic molecular changes. This holistic approach enables a more comprehensive understanding of the disease process, potentially leading to more accurate predictions of metastasis and patient outcomes. In the present study, we confirmed that four metastasis-related pathways were significantly annotated in several modules. The PPI network, which consisted of genes mapped to pathways known to be significantly related to metastasis through genetic analysis, showed good performance in predicting lymph node metastasis in NSCLC. Hypoxia is a major factor that promotes metastatic progression [31]. Hypoxia causes tumor invasion and metastasis via multiple mechanisms, including epithelial-mesenchymal transition [32]. Univariate analysis reveals that elevated levels of hypoxia-inducible factor 1α (HIF-1α) expression are significantly linked to lower disease-free survival rates in patients with NSCLC [33]. Additionally, this expression is correlated with survival rates, influencing the suppression of apoptosis [34]. Central carbon metabolism in cancer metastasis involves distinct metabolic adaptations and strategies employed by cancer cells to invade, survive, and grow at secondary sites [35]. Focal adhesions are specialized structures in the cell membrane where clusters of integrins mediate attachment between cells and the extracellular matrix. The focal adhesion pathway is crucial for various cellular processes, including cell migration, proliferation, differentiation, and survival [36,37,38]. The ubiquitin-proteasome system (UPS) is a critical regulator of many cellular processes, and its dysregulation has profound implications in cancer biology, including metastasis [39]. The UPS regulates the degradation of HIF-1 α, a critical factor in angiogenesis [40]. Tumor characteristics can be extracted through these genetic expression phenomena and image features, and these results increase the predictive power of the model.

While our study made significant strides in predicting lymph node metastasis in NSCLC using a GNN model, it is essential to note that our findings are based on a relatively modest sample size. This limitation inherently restricts the generalizability and robustness of our conclusions, underscoring the need for further validation of our model in larger and more diverse patient cohorts. The pursuit of larger datasets in future studies is not merely a matter of increasing numbers but also of enhancing the model’s ability to capture a broader spectrum of patient demographics, disease stages, and genetic variations. Such expansion would not only solidify the reliability of the model but also potentially reveal more nuanced insights into the complex interplay between genomic features and imaging data in the context of NSCLC. Future research should focus on addressing the current limitations through larger, more diverse datasets, exploring a variety of advanced deep learning techniques, and broadening the scope of validation to include various patient populations and potentially other cancer types. Such efforts will be instrumental in refining the predictive power of our model and maximizing its contribution to personalized medicine in oncology.

The integration of advanced imaging techniques with an intricate gene network analysis using a GNN model holds considerable promise for predictive oncology. Our study underscores the potential of this approach and sets the foundation for more comprehensive research. It demonstrates the power of combining deep learning techniques with comprehensive radiogenomic data, opening new avenues for diagnostic and therapeutic advancements in oncology. As the field continues to evolve, studies like this will be instrumental in guiding the development of more effective, personalized cancer care.

## 4. Materials and Methods

### 4.1. Data Sources

The ^18^F-fluorodeoxyglucose positron emission tomography/computed tomography (^18^F-FDG PET/CT) images of NSCLC patients were obtained from The Cancer Imaging Archive (http://doi.org/10.7937/K9/TCIA.2017.7hs46erv, accessed on 16 November 2023). The ^18^FDG-PET/CT images were obtained from 211 NSCLC patients recruited between 7 April 2008 and 15 September 2012 at the Stanford University Medical Center and Palo Alto Veterans Affairs Healthcare System. A GE Discovery ^18^FDG-PET/CT scanner was used to construct images before surgical treatment. The ^18^FDG-PET images were generated at both institutions using a similar protocol. Patients exhibiting distant metastasis or without accessible RNA expression data were omitted from the study. For the construction of the model data, 93 patients were incorporated contingent upon the following criteria: (a) a pathological diagnosis confirming the absence of regional node metastasis (n = 73), or (b) a pathological diagnosis identifying metastasis to the ipsilateral axillary, mediastinal, pulmonary, hilar, or infra-sub carinal nodes (n = 20).The basic and clinical characteristics of the patients in the internal and external cohorts are shown in Table 2.

### 4.2. Gene Analysis

Hub gene analysis was performed to identify metastasis-related genes and construct a PPI network. To identify the genes in the modules of highly correlated genes, weighted gene co-expression network analysis (WGCNA) was performed with 22,126 genes using the R (version 4.2.2) software (R Foundation for Statistical Computing, Vienna, Austria). Genes with differences below a certain threshold were removed using the WGCNA filtering function, and those showing significant changes across samples were analyzed. Outlier samples were removed using the sample clustering function, and a soft threshold power of 5 was selected to determine strong correlations between genes. Genes strongly associated with lymph node metastasis were identified by calculating the high gene significance. Because the modules correspond to biological pathways that are involved in biological processes, the functions of the genes within the modules were determined. Gene ontology (GO) term analysis was performed using the DAVID online application (https://david.ncifcrf.gov/summary.jsp, accessed on 16 November 2023). Metastasis-related pathways with a *p*-value < 0.05 were identified. Differentially expressed genes (DEGs) between lymph node metastasis and non-metastasis groups were determined using PathfindR in R (version 4.2.2) software (R Foundation for Statistical Computing, Vienna, Austria), with significance marked at a *p*-value < 0.05. Univariate analysis of genes was performed using the MedCalc (version 20.106) software (MedCalc Software, Mariakerke, Belgium) to describe the distribution and patterns of metastasis as a single variable. The PPI network was constructed using STRING (https://string-db.org, accessed on 16 November 2023) of the genes in each module and the selected hub genes.

### 4.3. Image Feature Extraction and Selection

Image features were extracted from the ^18^FDG-PET/CT images by using Local Image Features Extraction (version 4.90) software (http://www.lifexsoft.org). The tumor region of interest was drawn using a semi-automated segmentation method with a threshold standardized uptake value (SUV) of 2.0. Lesions were detected in the regions with elevated ^18^F-FDG uptake, as determined by the pathological contrast observed in the CT images. Subsequently, area under the curve receiver operating characteristic curve (AUC) values were calculated through the MedCalc (version 20.106) software (MedCalc Software, Mariakerke, Belgium). The statistical significance of the AUC values was ascertained using the Hanley and McNeil method, implemented. This involved calculating the standard error of each AUC, taking into account the number of positive and negative cases. Utilizing the Hanley and McNeil formula, which integrates the AUC values with case distribution, we computed the standard error. Z-statistics were then derived by comparing the AUCs to a null hypothesis value of 0.5 and dividing by the standard error. The resulting *p*-values, obtained from the z-statistics through the standard normal distribution, provided a measure of statistical significance. To reduce the risk of overfitting and bias stemming from variables with excessively high AUC values, we established a threshold of 0.68. Image features with AUC values above this threshold were chosen, as further analysis indicated that setting thresholds substantially lower or higher than 0.68 posed difficulties in model construction.

### 4.4. Prediction Model

**Figure 3:**
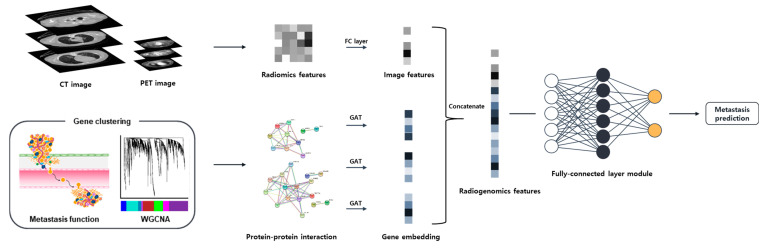
Scheme of the non-small cell lung cancer lymph node metastasis prediction.

**Figure 4:**
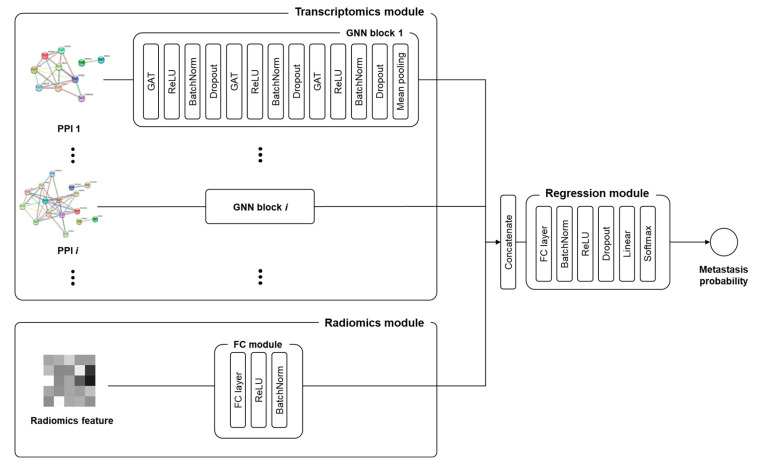
Structure of the graph neural network model for protein–protein interaction module characteristic recognition.

This study employed a GNN integrated with the synthetic minority over-sampling technique (SMOTE) to effectively manage sample imbalance, thereby enhancing the sample size to 146 individuals for more robust data analysis. SMOTE algorithm of the Scikit-learn library employs the k-nearest neighbors algorithm to generate new data points based on the characteristics of those that were underrepresented. Figure 3. shows a schematic of the deep learning prediction model used in this study. For effective extraction of groupwise transcriptomic features, a graph attention network (GAT) was used as the GNN layer. The GAT is a graph convolutional network-based layer in which a trainable masked attention mechanism is applied for better information propagation. In this study, we used three-head attention, and the output of the layer was calculated as the average. There were three GNN blocks, consisting of a GAT layer, rectified linear unit (ReLU), batch normalization, and dropout. The outcome, which was node embedding for each PPI, was transformed into graph-wise embedding by max pooling. The image features were also transformed by a fully connected (FC) layer module, which was composed of an FC layer, ReLU, and batch normalization, before being used as input for the classifier. The input of the classifier was obtained by concatenating several graph-wise PPI embeddings and transformed image features. The classifier was composed of two FC layers and a softmax layer to calculate the probability of metastasis (Figure 4). In this study, k-fold cross-validation were utilized, where the dataset was randomly divided into K subsets. During this process, each subset was used once as a test dataset while the rest formed the training dataset, repeated k-times to ensure robustness. The model was trained on Intel Xeon silver 4214R CPU (Intel, Santa Clara, CA, USA) and NVIDIA Titan V GPU (NVIDIA, Santa Clara, CA, USA).

## 5. Conclusions

The combination of ^18^F-FDG PET and CT image data improved the performance of the GNN model in various gene-related analyses. The predictive capability of the model was enhanced when a broader set of image texture features was considered. The GNN model developed in this study has potential clinical applications. Imaging features correlated with hub genes can be obtained in a noninvasive manner to enable early prediction of lymph node metastasis, which can help in the early establishment of treatment strategies in NSCLC. The development and validation of this innovative GNN model marks a pivotal step forward in our ability to offer more personalized, effective, and noninvasive care to patients suffering from this challenging disease.

## Figures and Tables

**Table 1:** Prediction results of lymph node metastasis in patients with non-small cell lung cancer using the GNN model.

	Metrics	Without Image Data	Image Features with AUC ≥ 0.68			Whole Image Features		
			PET (6)	CT (4)	PET/CT (10)	PET (55)	CT (55)	PET/CT (110)
73 modules *	Accuracy	0.4795	0.5375	0.5473	0.6420	0.7179	0.7429	0.8455
	Precision	0.2679	0.4390	0.5448	0.7633	0.7039	0.7000	0.8392
	Recall	0.4518	0.7750	0.6321	0.6696	0.8268	0.9018	0.9054
	F1 score	0.3248	0.5496	0.5219	0.6354	0.7439	0.7820	0.8631
	AUC	0.5674	0.5744	0.6532	0.7430	0.7728	0.7907	0.8718
Without gene data	Accuracy	-	0.5920	0.6857	0.7607	0.8357	0.8009	0.8286
	Precision	-	0.5515	0.6627	0.7662	0.7875	0.7565	0.8026
	Recall	-	0.7625	0.8125	0.8000	0.9339	0.9161	0.8893
	F1 score	-	0.6363	0.7199	0.7630	0.8522	0.8236	0.8812
	AUC	-	0.6549	0.7415	0.8035	0.8530	0.8372	0.8812
Genes in 4 functions (34) **	Accuracy	0.5830	0.6509	0.6018	0.7330	0.8143	0.7955	0.8545
	Precision	0.4538	0.6037	0.5858	0.7004	0.7928	0.7548	0.8213
	Recall	0.4036	0.6946	0.6018	0.8107	0.8768	0.9304	0.9143
	F1 score	0.3692	0.6377	0.5805	0.7477	0.8267	0.8238	0.8618
	AUC	0.6333	0.6776	0.7064	0.7709	0.8901	0.8849	0.9026
DEG results (19) ***	Accuracy	0.5411	0.6321	0.6429	0.6973	0.8000	0.7768	0.8223
	Precision	0.5239	0.6131	0.6146	0.6916	0.7553	0.7343	0.8006
	Recall	0.5875	0.6464	0.7357	0.7250	0.9179	0.9179	0.8893
	F1 score	0.4910	0.6153	0.6421	0.6963	0.8238	0.8098	0.8345
	AUC	0.5231	0.6879	0.7092	0.7458	0.8585	0.8245	0.8948
Univariate analysis (12) ****	Accuracy	0.5036	0.6045	0.7268	0.7580	0.8134	0.7580	0.8420
	Precision	0.3998	0.6098	0.6861	0.7443	0.7660	0.7256	0.7893
	Recall	0.5893	0.6750	0.8625	0.7982	0.9179	0.8464	0.9607
	F1 score	0.4627	0.5929	0.7602	0.7668	0.8325	0.7643	0.8610
	AUC	0.4784	0.7229	0.7627	0.7765	0.8606	0.8397	0.8793

* 73 modules: The construction of the GNN model in this study was based on 73 gene modules, which were identified through WGCNA. ** Genes in 4 functions: For the development of the GNN model, genes associated with four specific functions related to metastasis were utilized. *** DEG results: Genes identified through DEG analysis were employed in the construction of the GNN model. **** Univariate Analysis: In the construction of the GNN model, genes that were identified using univariate analysis were selectively employed.

**Table 2:** Clinical data of patients with non-small cell lung cancer.

Characteristics	Non Metastasis (n = 73)	Lymph Node Metastasis (n = 20)
Age (%)		
<65	22 (30)	3 (15)
≥65	51 (70)	17 (85)
Mean age (y)	68.82	69.1
Sex, n (%)		
Male	56 (77)	15 (75)
Female	17 (23)	5 (25)
Pathological stage, n (%)		
T1a	15 (21)	1 (5)
T1b	15 (21)	7 (35)
T2a	26 (36)	5 (25)
T2b	6 (8)	3 (15)
T3	7 (10)	2 (10)
T4	4 (5)	2 (10)
Pathological stage (%)		
N0	73	
N1		7 (35)
N2		13 (65)
Pathological stage (%)		
I	56 (77)	
II	13 (18)	7 (35)
III	4 (5.48)	12 (60)
IV		1 (5)

## Data Availability

The data supporting the findings of this study are available from the corresponding author on request; however, restrictions apply to the availability of the data that were used under license for the current study and are not publicly available.

## Supplementary Materials

The following supporting information can be downloaded at: https://www.mdpi.com/article/10.3390/ijms25020698/s1.
