# Supplementary material for: Graph Neural Network Model for Prediction of Non-Small Cell Lung Cancer Lymph Node Metastasis Using Protein–Protein Interaction Network and 18F-FDG PET/CT Radiomics
Source: Int J Mol Sci. 2024 Jan 5;25(2):698. doi: 10.3390/ijms25020698 (PMC10815846; doi:10.3390/ijms25020698)

Supplementary Materials

Supplementary Figure S1: Associations between gene modules and lymph node metastasis traits

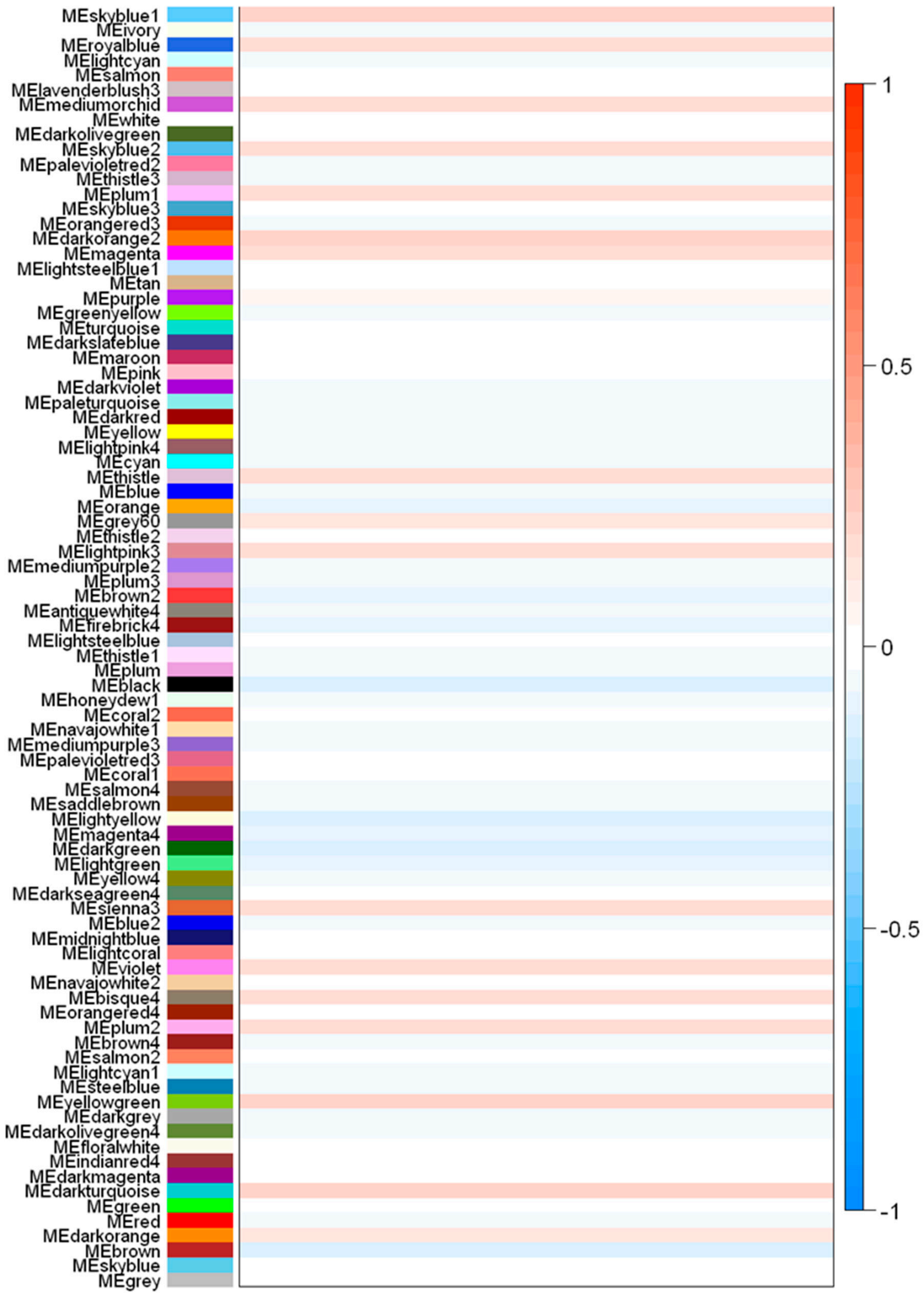

Supplement: Supplementary file 1 [file ijms-25-00698-s001.zip › ijms-2751246-supplementary.pdf]
